# Epigenetic and post-translational modifications in autophagy: biological functions and therapeutic targets

**DOI:** 10.1038/s41392-022-01300-8

**Published:** 2023-01-16

**Authors:** Feng Shu, Han Xiao, Qiu-Nuo Li, Xiao-Shuai Ren, Zhi-Gang Liu, Bo-Wen Hu, Hong-Sheng Wang, Hao Wang, Guan-Min Jiang

**Affiliations:** 1 Department of Clinical Laboratory, The Fifth Affiliated Hospital of Sun Yat-sen University, Zhuhai, Guangdong China; 2 Cancer Center, Affiliated Dongguan Hospital, Southern Medical University, Dongguan, Guangdong China; 3 Department of Urology, The Fifth Affiliated Hospital of Sun Yat-sen University, Zhuhai, Guangdong China; 4 Guangdong Key Laboratory of Chiral Molecule and Drug Discovery, School of Pharmaceutical Sciences, Sun Yat-sen University, Guangzhou, Guangdong China; 5 Department of Clinical Laboratory, The First Affiliated Hospital of USTC, Division of Life Sciences and Medicine, University of Science and Technology of China, Hefei, Anhui China

**Keywords:** Molecular medicine, Epigenetics

## Abstract

Autophagy is a conserved lysosomal degradation pathway where cellular components are dynamically degraded and re-processed to maintain physical homeostasis. However, the physiological effect of autophagy appears to be multifaced. On the one hand, autophagy functions as a cytoprotective mechanism, protecting against multiple diseases, especially tumor, cardiovascular disorders, and neurodegenerative and infectious disease. Conversely, autophagy may also play a detrimental role via pro-survival effects on cancer cells or cell-killing effects on normal body cells. During disorder onset and progression, the expression levels of autophagy-related regulators and proteins encoded by autophagy-related genes (ATGs) are abnormally regulated, giving rise to imbalanced autophagy flux. However, the detailed mechanisms and molecular events of this process are quite complex. Epigenetic, including DNA methylation, histone modifications and miRNAs, and post-translational modifications, including ubiquitination, phosphorylation and acetylation, precisely manipulate gene expression and protein function, and are strongly correlated with the occurrence and development of multiple diseases. There is substantial evidence that autophagy-relevant regulators and machineries are subjected to epigenetic and post-translational modulation, resulting in alterations in autophagy levels, which subsequently induces disease or affects the therapeutic effectiveness to agents. In this review, we focus on the regulatory mechanisms mediated by epigenetic and post-translational modifications in disease-related autophagy to unveil potential therapeutic targets. In addition, the effect of autophagy on the therapeutic effectiveness of epigenetic drugs or drugs targeting post-translational modification have also been discussed, providing insights into the combination with autophagy activators or inhibitors in the treatment of clinical diseases.

## Introduction

Autophagy is stimulated by cellular or environmental stresses through the formation of autophagosomes to clear damaged organelles, protein aggregates, and intracellular pathogens. Autophagy is a distinct biological process that exerts significant roles in determining the cellular status and fate. Autophagy orchestrated by ATGs is a conserved lysosomal degradation pathway where cellular components are dynamically degraded and re-processed to maintain physical homeostasis. However, the physiological effect of autophagy tends to be multifaced. On the one side, autophagy supports stress adaption and targets pernicious cargos, such as damaged protein aggregates, dysfunctional organelles, and invasive pathogens.^1,2^ This cytoprotective mechanism protects against multiple diseases, especially tumor, cardiovascular disorder, and neurodegenerative and infectious disease. Conversely, autophagy can also display a detrimental role via pro-survival effects on cancer cells or cell-killing effects on normal body cells.^3^

Epigenetic and post-translational modifications, respectively, manipulate gene expression at transcriptional and post-transcriptional level, and affect protein activity, function and degradation at post-translational level. These modifications are highly critical for normal cellular development and maintenance of tissue-specific gene and protein expression, a dysregulation of which will cause abnormal genes and protein expression signature and malignant phenotype transformation that induces disease occurrence and progression.^4,5^ The epigenetic and post-translational regulation of autophagy is very complex and plays different roles in human disease. Under certain circumstances, autophagy-related regulators and components, such as TFs, ATG proteins and signaling effector proteins undergo epigenetic and post-translational regulation, leading to autophagy activation or inhibition. Therefore, the clinical outcome of autophagy in different diseases is still a mystery and whether autophagy represses and promotes disease development remains deep investigation.

Considering the scarcity of knowledge in terms of role of epigenetic and post-translational alteration of autophagy in human diseases, the present review attempts to understand and summarize the effects of epigenetic and post-translational modifications of autophagy-related regulators and components in human disease. It will be conductive for uncovering the underlying regulatory mechanisms of disease-related autophagy, seeking for new targets for better diagnosis and treatment. Meanwhile, due to the prospective exploration and application of epigenetic drugs and some drugs targeting post-translational modifications, the different effects of autophagy following these drugs treatment in human diseases are also reviewed here, providing insights into the combined application with autophagy activators or inhibitors.

### Autophagy and its main regulatory mechanism

The basic autophagy process, orchestrated by series of autophagy-related regulators and ATG-encoded downstream effector proteins, can be divided into four predominant steps: autophagy initiation (containing ULK1 complex-ULK1/FIP200/ATG13), vesicle nucleation, elongation and autophagosome formation (containing Beclin1-VPS34 complex, ATG5/ATG12/ATG7/ATG10/ATG16L, LC3/ATG3/ATG7/ATG4 and p62, OPTN, NIX), autophagosome-lysosome fusion (containing Rab GTPases and *N*-ethylmaleimidesensitive factor attachment protein receptors (SNAREs)), and cargo degradation.^6^

The autophagy process is induced by cellular or environmental stimulus, such as nutrient deprivation, metabolic and energetic stresses, pathogen invasion or oxidant stresses. mTOR subtly senses intra- and extra-cellular nutrient and stress levels, and its phosphorylation and activation suppress the autophagy process by blocking ULK1 complex activity. PI3K/AKT/mTOR and AMPK/mTOR are two quite significant upstream signaling pathways of mTOR in autophagy regulation, which is implicated in tumor progression, myocardial dysfunction, neurodegeneration and metabolic disorders.^7,8^ Moreover, Wnt signaling pathway is associated with autophagy repression, and is involved in embryogenesis and differentiation. During nutrient deprivation, β-catenin and Dishevelled proteins are targeted for autophagic degradation by LC3. When Wnt signaling is activated, β-catenin acts as a corepressor of p62 to inhibit autophagy. In contrast, another key Wnt signaling protein, GSK3β, negatively regulates the Wnt pathway and has been shown to induce autophagy by phosphorylation of the TSC complex.^9^ Under ER stress, the unfolded protein response (UPR) has been found to activate ATGs expression and subsequent autophagy, which is mediated by ATF4-CHOP and TRAF2-IRE1-JNK signaling pathway. ATF4 and CHOP transcriptionally regulates dozens of ATG genes and TRAF2-dependent activation of IRE1 and JNK results in Bcl2 phosphorylation, enables the dissociation of Beclin-1.^10^

### Autophagy and diseases

**Fig. 1:**
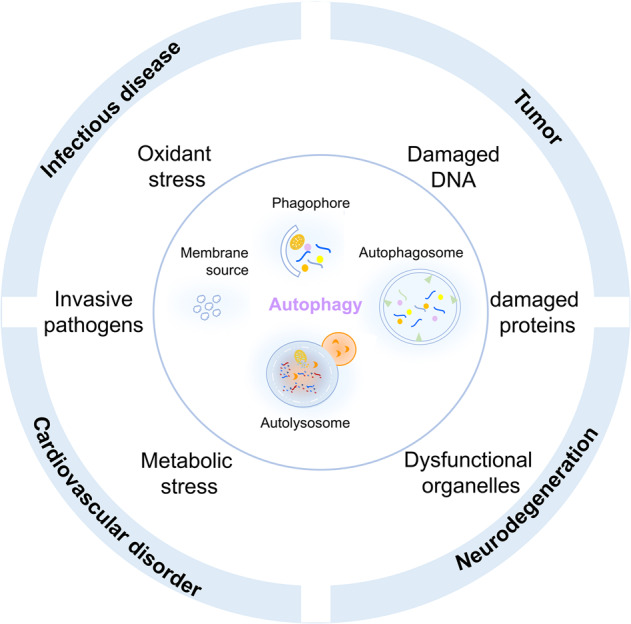
Autophagy and diseases. Autophagy dysregulation and tumor, inflammatory disease, neurodegenerative disease, cardiovascular disease

Over the past few years, the relationship between autophagy dysregulation and multiple human diseases has been extensively investigated and appears to be quite complicated.^2^ Taking advantage of gene-knockout technology in vitro experiments and animal models, research studies have confirmed a set of autophagy-related genes to be closely linked to diseases, particularly tumor, inflammatory disease, neurodegenerative disease, cardiovascular disease, these autophagy-related genes play key roles in human diseases, even though autophagy-relevant detection in humans remains difficult in clinical tests (Fig. 1).

In terms of autophagy and tumor occurrence and progression, autophagy seems to be a ‘double-edged sword’ and its roles is mainly dependent on specific tumor types, stages, and environmental stresses. Great efforts have been made to decode their connecting mechanisms from different perspectives, including crosstalk between autophagy and tumor metabolic alteration,^11^ autophagy and antitumor or tumor-promoting immune responses.^12^ During the tumor initiation period, most models regard autophagy as an antitumor mechanism, and a few essential autophagy-related proteins and transcriptional factors, such as Beclin1, PARK2 (Parkin), and Forkhead box O (FOXO) family proteins, have been implicated as tumor suppressors that impede further malignant transformation.^13–15^ Nevertheless, along with tumor progression, autophagy can serve as pro-survival mechanism to assist tumor cells adaption to metabolic and energetic stresses.^11,16,17^ One typical case is the transformation of the roles of autophagy from antitumor autophagy to tumor-promoting autophagy within primary melanoma and metastatic melanoma.^18^ Since neurodegenerative diseases are characterized by the accumulation of abnormal protein condensates and aggregates (e.g., tau, APP, SOD1, α-synuclein, and polyglutamine proteins), defect of autophagic degradation can lead to clearance impediment and is one of the reasons for neurodegeneration. For instance, Beclin1 deficiency is prevalent in Alzheimer’s disease (AD) and Huntington disease.^19,20^ PICALM acts as an autophagy receptor that is able to interact with LC3 and target APP into autophagosomes, and PS-1 functions as an ER chaperone for the V_Oa1_ subunit of the lysosomal v-ATPase and thus lysosomal PH maintenance, whereas PICALM and PS-1 mutations are frequently occurred in AD patients.^21,22^ In response to oxidative stress, Nrf2 induces autophagy receptor NDP52 to stimulate autophagy and remove aggregated tau proteins.^23^ ATP13A2 involved with lysosomal ATPase, is found mutated in autosomal recessive forms of early-onset Parkinsonism to accelerate α-synuclein accumulation.^24^ Autophagy is also involved in the regulation of diabetic complications, such as diabetic nephropathy, renal fibrosis and cardiomyopathy. mTOR/AMPK, TFEB/ZNSCAN3, SIRTs and FOXOs establish links between autophagy and diabetic complications.^25,26^

## Epigenetic modifications and autophagy-related diseases

Epigenetic modifications including DNA methylation, histone modification and microRNAs play important roles in autophagy-related diseases. DNA methylation and covalent modifications of histone protein are dynamically orchestrated by a so-called series of epigenetic enzymes known as “writers”, “readers” and “erasers” to control and fine-tune large-scale gene transcription.^27,28^ microRNAs mainly via base pairing binds to gene mRNA to regulate gene post-transcriptional process.

### DNA methylation regulates ATGs expression

DNA methylation refers to the addition of a methyl group at the 5-position cytosine (5-methylcytosine (5mC)) in CpG dinucleotides of the genome. This process is catalyzed by a family of DNA methyltransferases (DNMTs), including DNMT1, DNMT3A, DNMT3B, and DNMT3L. Aberrant DNA methylation and demethylation events sustain autophagy level at an abnormal inactivation or activation status in multiple disease, such as tumor and nervous system lesion. A cohort of studies have discovered a series of disease-relevant DNA methylation events linked to autophagy deficiency and hyperactivation, conducive to exploring potent therapeutic targets and prospective epigenetic markers for prognosis. (Table 1).

**Table 1:** DNA methylation of autophagy-related genes in diseases

Autophagy-related genes	Methylation status	Disease types	Autophagy activation or inhibition	The effect on diseases
MAP1LC3Av1	Methylation	Gastric carcinoma^29^	Inhibition	Tumor promotion
ATG4A	Hypomethylation	Ovarian cancer^30^	–	Tumor promotion
ATG16L2	Methylation	Childhood acute lymphoblastic leukemia^31^	Activation	Poor prognosis in response to imatinib treatment
Beclin1	Methylation	Breast cancer^32^	Activation	Tamoxifen resistance
GABARAPL1	Methylation and histone deacetylation	Breast cancer^33^	Inhibition	Tumor promotion
ATG2B/ATG4D/ATG9A/ ATG9B	Methylation	Ductal breast carcinomas^34^	Inhibition	Tumor invasion
ATG13/DNA damage-regulated autophagy modulator protein 1	Methylation	Glioma cells^36^	Inhibition	Tumor promotion
BNIP	Demethylation	Esophageal squamous cell carcinoma^42^	Activation	Tumor pro-survival
ATG16L	Methylation	Medulloblastoma^43^	Inhibition	Tumor suppression
ALP genes	Methylation	Parkinson disease^44^	Inhibition	α-synuclein aggregation
–	Methylation	Huntington disease^45^	Inhibition	Cytotoxicity
LC3	Methylation	aging-related diseases^46^	Inhibition	Aging

It was reported that *Helicobacter pylori*-induced MAP1LC3Av1 methylation silencing leads to autophagy impairment and promotes gastric carcinogenesis.^29^ ATG4A is hypomethylated in ovarian tumor-initiating cells and predicts poor prognosis in ovarian cancer patients.^30^ Frequently hypermethylated ATG16L2 in childhood acute lymphoblastic leukemia is associated with a poor prognosis in response to imatinib treatment.^31^ Reduced Beclin1 promoter methylation due to DNMT3B inhibition increases autophagy and promotes tamoxifen resistance in breast cancer.^32^ High GABARAPL1 expression is a suitable prognostic marker in lymph node-positive breast cancer patients. DNA methylation and histone deacetylation cause its downregulation in breast cancer.^33^ Similarly, increased levels of methylated ATG2B, ATG4D, ATG9A, and ATG9B have also been observed in invasive ductal breast carcinomas. ATG2B methylation is positively correlated with tumor grade and stage, whereas ATG4D and ATG9A methylation is positively linked to lymph node involvement.^34^ Ten-eleven translocation (TET) proteins, such as 5mC oxidases, are crucial in active DNA demethylation through iterative methyl group oxidation, completing the conversion of 5mC into 5-hydroxymethylcytosine (5hmC). TET1 exerts its tumor suppressor function by regulating autophagy.^35^ Due to reduced TET1 expression in glioma cells, two ATGs, ATG13 and DNA damage-regulated autophagy modulator protein 1, display low 5hmC enrichment in their promoter region, leading to lower autophagy levels than in normal controls.^36^ Hypoxia can induce autophagy, hypoxia-induced interleukin-6 (IL6) acts as an autophagy initiator for tumor-promoting effects,^37^ hypoxia-induced astrocyte elevated gene-1 (AEG-1) regulates autophagy in the drug resistance of T-cell non-Hodgkin’s lymphoma,^38^ and hypoxia-inducible factor^39^ family proteins, which are hallmarks of hypoxia, activate autophagy through elevated ATG expression.^40,41^ Under hypoxic conditions, decreased BNIP methylation induces pro-survival autophagy in esophageal squamous cell carcinoma (ESCC),^42^ whereas HIF1A knockdown increases the methylation of ATG16L to limit medulloblastoma cell proliferation.^43^

Furthermore, autophagy-lysosome pathway (ALP) is frequently disrupted in Parkinson disease, leading to α-synuclein aggregation. It has identified aberrant methylation at 928 cytosines affecting 326 ALP genes in the appendix of individuals with PD and widespread hypermethylation that is also seen in the brain of individuals with PD.^44^ DNMT1 acts on neurodegeneration by modulating proteostasis-relevant intracellular processes. DNMT1 negatively impacts retrograde trafficking and autophagy, which involved in the clearance of aggregation-prone proteins by the aggresome-autophagy pathway. In Huntington disease, DNMT1 knockdown strengthens autophagy, and ameliorates Huntington-induced cytotoxicity.^45^ Decrease of autophagy activity is occurring upon aging and thought to contribute to aging-related diseases. It uncovers, upon autophagy induction, de novo DNMT3A-mediated DNA methylation on expression of LC3 results in reduced baseline autophagy activity.^46^ Meanwhile, ULK3-dependent activation of GLI1 contributes to upregulation of DNMT3A gene expression upon autophagy induction, thereby bringing additional understanding of the long-term effect of autophagy induction and a possible mechanism for its decline upon aging, pathological conditions, or in response to treatment interventions.^47^

### Histone methylation

#### G9a/EHMT2

**Fig. 2:**
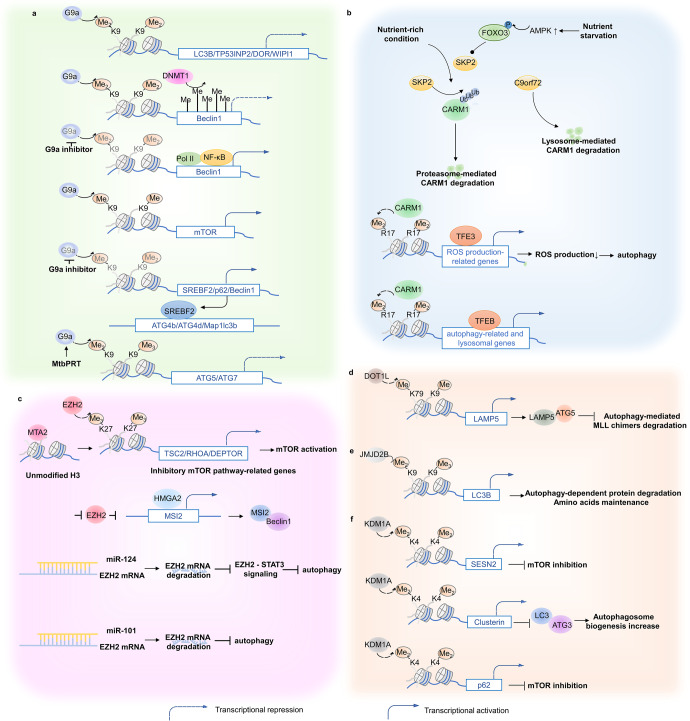
Histone methylation and autophagy regulation. **a** G9a negatively regulates core autophagy effectors and upstream autophagic regulators to influence autophagy level indirectly. **b** Under a nutrient-rich environment, SKP2 mediates CARM1 protein degradation, nutrient starvation activates AMPK-dependent FOXO3 phosphorylation, which transcriptionally represses SKP2, resulting in CARM1-mediated transcriptional activation of autophagy-related and lysosomal genes. **c** EZH2 repressively regulates autophagy via mTOR activation. EZH2 negatively controls TSC2/RHOA/DEPTOR gene transcription to elicit mTOR pathway, leading to autophagy inhibition. Additionally, EZH2 represses HMGA2 expression. HMGA2 can directly activate the MSI2 promoter region, which triggers autophagy via Beclin1 interactions. **d** DOT1L elevates LAMP5 expression via H3K79 methylation modification enhancements. LAMP5 directly interacts with ATG5 to interrupt an autophagy flux, protecting MLL chimeras from autophagy degradation. **e** JMJD2B promotes autophagy occurrence via histone demethylation at LC3B promoters, assisting in intracellular amino acid maintenance. **f** KDM1A negatively manipulates autophagy flux through SESN2- and CLU-dependent pathways or directly targeting p62. SESN2 inhibits mTORC1 activity, and CLU increases autophagosome biogenesis through MAP1LC3/LC3-ATG3 heterodimer stabilization

G9a/ Euchromatic histone lysine methyltransferase 1 (EHMT2) catalyzes methylation of histone 3 at lysine 9 (H3K9me1, H3K9me2, and H3K9me3) and is frequently overexpressed in various cancers, including glioma, gastric cancer, and lung cancer.^48–50^ G9a/EHMT2 overexpression was significantly associated with autophagy suppression. It can directly interact with the promoters of core autophagy genes, such as LC3B, TP53INP2/DOR, and WIPI1, increasing their histone methylation levels to reduce autophagy levels.^51^ In particular, in breast cancer cells, G9a cooperates with DNMT1 to collectively inhibit Beclin1 transcription.^52^ Chemical inhibition of G9a dissociates H3K9me2 from the Beclin1 promoter and recruits RNA polymerase II and nuclear factor kappa B (NF-κB) to its promoter for transcriptional activity. When G9a is inhibited, BNIP3 expression is transcriptionally activated, releasing Beclin1 from the B-cell lymphoma 2 (Bcl-2)/Beclin1 complex to trigger autophagy.^53^ In addition, mTOR is a direct target that is monomethylated by G9a at H3K9 and transcriptionally activated.^49^ It is reasonable to determine the connection between G9a expression, cellular nutritional status, and autophagy for mTOR’s role in regulating autophagy in response to integrated signals from changed nutrient and energy levels. In non-small cell lung cancer (NSCLC), EHMT2 inhibition leads to cancer cell death via autophagy induction, in which sterol regulatory element-binding protein 2 (SREBF2) expression is a crucial contributor.^54^ SREBF2 can communicate with the autophagy process by binding to the promoters of several autophagy genes, such as Map1lc3b, ATG4b, and ATG4d.^55^ Here, EHMT2 inhibition robustly decreases H3K9me1 and H3K9me2 at the promoter of the SREBF2 locus, resulting in upregulated SREBF2 expression and, consequently, autophagy-mediated cell death. Similarly, G9a is capable of H3K9me/me2 on the promoter of Beclin1 and P62 to transcriptionally inhibit autophagy flux, and then induces vascular smooth muscle cells death, indicating that G9a may be a potent therapeutic target for cardiovascular diseases including aortic dissection.^56^ In addition, Mtb phosphoribosyltransferase (MtbPRT) induces EHMT2-dependent H3K9me2/3 and reduces H3K9ac and H3K27ac by upregulating HDAC3 at the ATG5 and ATG7 promoter, which inhibit autophagy to promote Mtb survival^57^ (Fig. 2a).

#### CARM1

CARM1 is responsible for histone H3 demethylation at arginine 17 (H3R17me2). The AMP-activated protein kinase (AMPK)-SKP2-CARM1 signaling pathway is involved in autophagy transcriptional regulation, and this has been found to pathologically associate with multiple diseases. CARM1 is normally degraded by S-phase kinase-associated protein (SKP) 2 (SKP2)-containing SKP1-cullin1-F-box protein (SCF) E3 ubiquitin ligase in the nucleus rather than in the cytoplasm in nutrient-rich environments. Conversely, nutrient starvation activates AMPK-dependent FOXO3 phosphorylation in the nucleus, transcriptionally repressing SKP2, which results in decreased CARM1 protein degradation. Upregulated CARM1 protein subsequently increases H3R17me2 on the promoters of autophagy-related and lysosomal genes, contributing to the transcriptional activation of these genes through the cooperative action of TFEB.^58^ In hepatocellular carcinoma (HCC), SKP2 levels were upregulated compared with corresponding normal liver tissues, which is statistically correlated with HCC progression.^59^ Moreover, during spinal cord injury, enhanced reactive oxygen species induce lysosomal dysfunction and then contributes to neuronal death, while TFE3 activation reverses outcomes of spinal cord injury, which is partly regulated by AMPK-SKP2-CARM1 signaling axis.^60^ In addition, C9orf72, linked with ALS-FTD, can promote the lysosomal degradation of CARM1, which in turn regulates autophagy-lysosome function and lipid metabolism.^61^ (Fig. 2b).

#### EZH2

EZH2, a catalytic subunit of polycomb repressive complex 2 (PRC2), mainly catalyzes the trimethylation of lysine 27 at histone H3 (H3K27me3) and is responsible for transcriptionally silencing target genes. On the one hand, EZH2 inhibition-triggered autophagy induces cell death of cancer cells^62,63^ and aortic vascular smooth muscle cells,^64^ liver injury^65^ and also helps bacterial elimination.^57^ In cancer cells, metastasis-associated 1 family member 2 (MTA2) first recognizes unmodified H3 with its SANT domain, then recruits EZH2 to a set of mTOR pathway-related genes, such as TSC2, RHOA, and DEPTOR, which are negative regulators of the mTOR signaling pathway, resulting in increased H3K27me3 accompanied by reduced H3K27ac at the promoters. As a result, the expression of mTOR pathway inhibitory genes is suppressed, which elicits mTOR activity and leads to autophagy inhibition.^66^ On the other hand, autophagy is triggered by EZH2 inhibition to serve as a possible pro-survival mechanism of residual tumor cells with high mobility group A2 (HMGA2) as the critical mediator.^67^ EZH2 is a repressive modulator of HMGA2. HMGA2 can directly activate the Musashi-2 (MSI2) promoter region, triggering autophagy via interactions with Beclin1, favoring cell proliferation.^68^ Recently, a few studies have revealed that a group of disease-related microRNAs cause alterations in autophagy levels by EZH2 regulation. For example, microRNA-638 and microRNA-92b increase autophagy and apoptosis and suppress cancer cell invasion and migration by targeting EZH2.^69,70^ miR-124 is capable of inducing autophagic cell death in tumors and alleviates autophagy-mediated diabetic peripheral neuropathy by directly targeting the EZH2-signal transducer and activator of transcription 3 (STAT3) signaling axis.^71,72^ In contrast, miR-101 directly interacts with EZH2 and downregulates its expression, resulting in autophagy inhibition^73,74^ (Fig. 2c).

#### DOT1L

DOTIL is an H3K79 histone methyltransferase. DOT1L expression is indispensable in mix-lineage leukemia (MLL) maintenance and progression by enhancing H3K79 methylation at its target gene loci, such as Meis1 and HOX gene clusters.^75,76^ The autophagic protein degradation pathway is repressed to sustain MLL fusion protein levels during MLL maintenance and progression. During this process, DOT1L targets the genetic locus of a lysosome-associated membrane protein family member (LAMP5) to enhance its H3K79 methylation modification to elevate its expression. Unlike other LAMP family members, such as LAMP1 and LAMP2, which act as enhancers in the autophagy pathway, LAMP5 directly interacts with ATG5, rather than Beclin1 or LC3, to interrupt an autophagy flux and protect MLL chimeras from autophagic degradation^77^ (Fig. 2d).

#### JMJD2B/ KDM4B

JMJD2B/KDM4B mainly reverses the tri- or demethylation (me3/2) of histone 3 at lysine 9. Epigenetic JMJD2B modifications stimulate autophagy levels and are linked to cancer cell proliferation. In CRC cells, JMJD2B is highly expressed and is accompanied by upregulated ATG signatures. Through direct demethylation at the promoters of LC3B, JMJD2B promotes autophagy to benefit protein degradation and recycling to maintain intracellular amino acids, which is critical for CRC cell survival under glucose deprivation conditions.^78^ Furthermore, in castration-resistant prostate cancer (CRPC), enhanced KDM4B depends on its demethylating activity to activate autophagy by regulating Wnt/β-catenin signaling, leading to CRPC cell proliferation^79^ (Fig. 2e).

#### LSD1/KDM1A

The histone demethylase LSD1/KDM1A manipulates autophagy directly regulating autophagy-related genes expressions, including ATGs, Beclin-1, LC3 and SQSTM1/p62, it also regulating the activities of some other autophagic signaling proteins such as p53, SESN2, mTORC1 and PTEN, which is implicated in tumors, vascular disorder and inflammation. KDM1A depletion triggers autophagy through the SESN2-dependent pathway.^80,81^ Mechanistically, KDM1A interacts with the promoter region of the SESN2 gene and represses its expression, whereas KDM1A inhibition or knockdown increases H3K4me2 levels, together with increased histone H3 acetylation and decreased H3K27me3, leading to SESN2 gene expression. SESN2, as a member of the SESN1/PA26-related protein family involved in cellular responses to different stress conditions, inhibits mTORC1 activity by regulating the Seh1-associated complex,^82^ leading to consequent autophagy induction.^83,84^ Another KDM1A regulatory approach in autophagy is that KDM1A, with MYCN, inhibits clusterin (CLU) transcription, which is an integral part of the autophagic process as it increases autophagosome biogenesis by stabilizing the MAP1LC3/LC3-ATG3 heterodimer.^85,86^ In addition, as for ovarian aging, LSD1 represses autophagy level by affecting H3K4me2 level and regulating the transcription of p62 to protect oocytes against preterm death^87^ (Fig. 2f).

### Histone acetylation

Histone acetyltransferases (HATs) and histone deacetylases (HDACs) are essential epigenetic enzymes that mechanistically manipulate the acetylation of both histone and non-histone proteins in a dynamic manner. The transcriptional silencing or activation is typically associated with the modified status and site on histone. HDAC inhibition or overexpression regulates autophagy pathway-related genes expression, promoting autophagy-mediated tumor progression and tissue injury.^28,88–90^

**Fig. 3:**
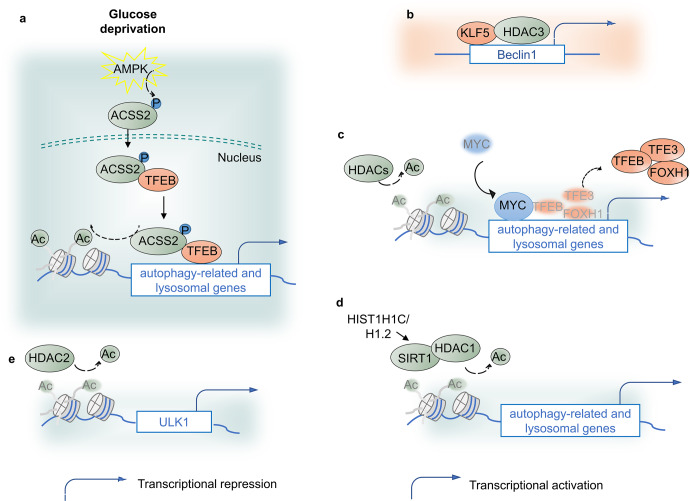
Histone acetylation and autophagy regulation. **a** Glucose deprivation assists AMPK-mediated ACSS2 phosphorylation and promotes its nuclear localization. In the nucleus, ACSS2 binds to TFEB and translocates to lysosomal and autophagy gene promoters, where ACSS2 generates acetyl-CoA for histone H3 acetylation and gene expression. **b** KLF5 protein and HDAC3 bind to the Beclin1 gene promoter and suppress its transcription, leading to autophagy suppression. **c** Coupled with MYC, HDACs, especially class I HDACs, epigenetically negatively regulate autophagic and lysosomal function. **d** HIST1H1C/H1.2 upregulates SIRT1 and HDAC1 to maintain the deacetylation of H4K16, leads to ATG proteins expression and then promotes autophagy, inflammation and cell toxicity. **e** Small-chain fatty acids treatment-reduced HDAC2 upregulates H3K27ac on ULK1 promoter, reduces ULK1 expression and autophagy flux and thus alleviate diabetic renal fibrosis

Acetyl coenzyme A (acetyl-CoA) is an important donor for the acetylation of proteins, and acetyl-CoA synthetase 2 (ACSS2) manipulates its expression. Glucose deprivation assists AMPK-mediated ACSS2 phosphorylation and promotes its nuclear localization. In the nucleus, ACSS2 binds to TFEB and translocates to lysosomal and ATG promoters, where ACSS2 generates acetyl-CoA for histone H3 acetylation and gene expression, promoting lysosomal biogenesis, autophagy, cell survival, and brain tumorigenesis.^91^ In castration-resistant prostate cancer (CRPC), the Kruppel-like factor 5 (KLF5) protein and HDAC3 collectively bind to the Beclin1 promoter and suppress its transcription, leading to autophagy suppression and increased drug sensitivity to docetaxel. Moreover, coupling MYC and HDACs, especially class I HDACs, such as HDAC2, negatively epigenetically regulates autophagic and lysosomal function by concomitantly affecting histone acetylation and gene expression of TFEB, TFE3, and FOXH1 TFs, as well as autophagic and lysosomal elements. MYC occupies the promoters of lysosome and ATGs, resulting in TFEB, TFE3, and FOXH1 promoter dissociation.^90^ As a classic tumor inducer, MYC is frequently overexpressed and genetically mutated in numerous human cancers.^92^ Therefore, it is likely to support the notion that tumors rely on the HDAC/MYC-TF axis to sustain proliferation and growth by repressing autophagic and lysosomal systems. HDACs is supportive in autophagy-induced diabetic retinopathy and renal fibrosis. In the development of diabetic retinopathy, overexpressed histone HIST1H1C/H1.2, an important variant of the linker histone H1, upregulates SIRT1 and HDAC1 to maintain the deacetylation of H4K16, leads to ATG proteins expression, and then promotes autophagy, inflammation and cell toxicity.^93^ Small-chain fatty acids treatment-reduced HDAC2 upregulates H3K27ac on ULK1 promoter, reduces ULK1 expression and autophagy flux and thus alleviate diabetic renal fibrosis^94^ (Fig. 3).

### MicroRNA

**Fig. 4:**
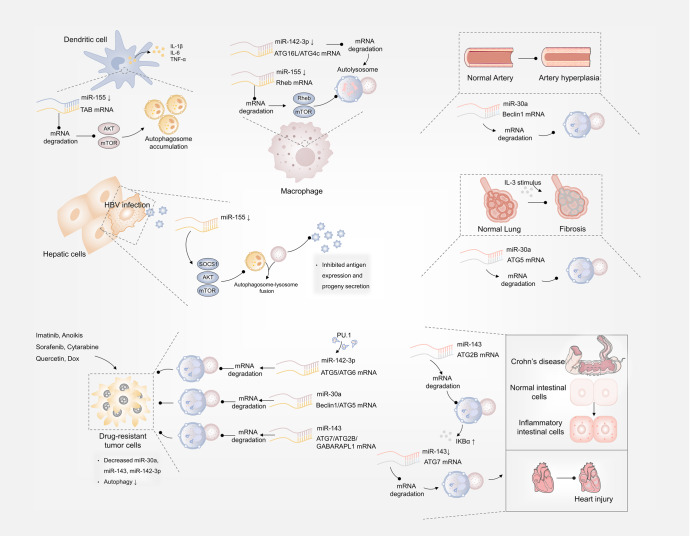
MicroRNA and autophagy regulation. In Behcet’s disease, HBV and mycobacterial infection, decreased miR-155 respectively inhibited TAB-AKT/mTOR-, SOCS1/Akt/mTOR- and Rheb/mTOR-dependent autophagy. miR-30a, miR-143 and miR-142-3p respectively targeted Beclin1/ATG5, ATG7/ATG2B and ATG5/ATG6 mRNA for degradation to inhibit autophagy and increase chemotherapy sensitiveness. miR-30a mediated autophagy suppression via targeting Beclin1 or ATG5 and alleviated arterial injury and airway fibrosis. Downregulated miR-142-3p could target ATG16L1, ATG4c to trigger autophagy and eliminate mycobacteria. miR-143 inhibited autophagy via targeting ATG7 or ATG2B, which induced Crohn’s disease or in other case, alleviated cardiac injury

MicroRNA (miRNA) is a major class of small noncoding RNA, which is quite conserved in biological evolution, typically blocks gene expression at a post-transcriptional level. The role of some specific miRNAs in regulating disease-related autophagy pathway have been revealed, and they mainly directly target autophagy-related components or signaling effector proteins to induce or inhibit autophagy process. There are a set of miRNAs that share in more than one disease and represent their universal pathogenic roles (Fig. 4).

miR-155-regulated autophagy implicates in viral hepatitis, Behcet’s disease and tuberculosis. miR-155 reinforces HBV replication by reinforcing the SOCS1/Akt/mTOR axis-stimulated autophagy.^95^ Decreased miR-155 in Behcet’s disease leads to defective autophagy in dendritic cells through Akt/mTOR pathway thereby stimulating cytokine production.^96^ After mycobacterial infection, enhanced miR-155 expression directly targets Rheb to accelerate autophagy process via inhibit Rheb/mTOR pathway for mycobacterial elimination.^97^ miR-30a-mediated autophagy suppression via targeting Beclin1 or ATG5 sensitizes tumor cells to chemotherapy,^98–100^ and alleviates airway fibrosis,^101^ arterial injury.^102^ Similarly, miR-142-3p could target ATG16L1, ATG4c or ATG5 to inhibit autophagy, promoting intracellular survival of mycobacterium tuberculosis or sensitizing hepatocellular carcinoma to sorafenib.^103,104^ Moreover, targeting ATG7, ATG2B or GABARAPL1, miR-143-inhibited autophagy promotes chemotherapy sensitivity of tumors, support cardiac progenitor cells survival and heart repair and increase inflammatory response in the development of Crohn’s disease.^105–108^

## Post-translational regulation and autophagy-related diseases

After transcripts are translated into proteins, PTMs participate in further processing of the protein products by inducing their covalent linkage to new functional groups, such as phosphate, ubiquitin, methyl groups, and acetate, which are critical for protein activity, stability and folding, protein co-interaction, and function execution.^109^ Various PTMs on autophagy-related components, TFs, and upstream effectors have been identified as essential autophagy-regulating approaches leading to the activation and suppression of autophagy process. Here, we summarize PTMs of autophagy process in disease conditions, and their implications for disease progression and repression, expecting the discovery of potential new targets (Table 2).

**Table 2:** Post-translational autophagy events in diseases

Post-translational event	Modified protein	Modified site or type	Autophagy activation or inhibition	Disease types	The effect on diseases
Acetylation	TFEB	K91, K103, K116, K430	Activation	Tumor^112^	Tumor suppression
	TFEB	K274, K279, GCN5-mediated	Inhibition	Neurodegeneration^113^	Neurodegeneration exacerbation
	P53	K120, Tip60-mediated	Activation	Tumor^114^	Tumor suppression
	P53	K382, SIRT1-mediated	Activation	Tumor^115^	Tumor suppression
	P53	K373, K382, p300/CBP-mediated	Activation	HCC^116^	Tumor suppression
	P53	SIRT1-mediated	Inhibition	sepsis-induced acute kidney injury^117^	Injury ↑
	FOXO1 (cytoplasm)	K262, K265, K274, SIRT2-mediated	Activation	Tumor^15,120^	Tumor suppression
	FOXO3a	HDAC4-mediaed	Inhibition	Vascular disorder^121^	Vascular inflammation ↓
	Beclin1	K430, K437, p300- and p300/SIRT1-mediated	Inhibition	Tumor,^124,125^ sepsis-induced acute kidney injury^126,127^	Tumor development
	LC3	SIRT1-mediated	Activation	Liver cancer^129^	Tumor development
	ATG5, ATG7	SIRT1-mediated	Activation	Liver cancer^130^	Chemoresistance
	STAT3/AKT	SIRT1-mediated	Activation	Adiposity^131^	Adipogenesis ↓
	STAT1	HDAC4-mediated	Activation	Diabetes^132^	Podocyte injury
	Cortactin/ tubulin	HDAC6-mediated	Inhibition	Juvenile-onset atypical Parkinson’s disease^134^	Protein aggregates accumulation
	Microtubules	HDAC6-mediated	Activation	Spinal cord injury^135^	Injury↓
	α-tubulin	HDAC6-mediated	Activation	Prostate cancer,^136^ Cockayne syndrome^137^	Tumor suppression, subcutaneous fat ↑
	α-tubulin/Tau	HDAC6-mediated	Activation	Neurological disorders^138^	/
Ubiquitination	ULK1	K63-linked, TRAF6-mediated	Activation	CML^140^	Tumor development
	Beclin1	K63-linked, TRAF6-mediated	Activation	Lung cancer^144^	Tumor development
				NSCLC^147^	Tumor suppression
	VPS34	K29, K48-linked	Inhibition	Steatosis^148^	Lipid metabolism balance
		KLHL20-mediated	Inhibition	diabetes-associated muscle atrophy^142^	–
	LC3B	TRAF6-mediated	Activation	Tumor^149^	Tumor development
	OPTN	K193 sites, K27, K48-linked, HACE1-mediated	Activation	Tumor^139^	Tumor suppression
	p62	K420 sites, K11-linked, SPOP-mediated	Inhibition	Prostate cancer^152,153^	Tumor development
Phosphorylation	ULK1	S757,S504 p38 MAPK-mediated	Inhibition	Neuroinflammatory diseases^154^	Inflammation ↑
	ULK1	S469, S495, S533, TOPK-mediated	Inhibition	Glioma^155^	Tumor suppression
		S405,S415 GSK3β-mediated	Activation	Pancreatic cancer^156^	Tumor development
	p62	S351, ULK1-mediated	Activation	ALS-FTLD^157^	Neurotoxicity
	ATG14	S29, ULK1-mediated	Activation	Huntington’s disease^158^	Polyglutamine disease protein clearance
	ATG5	T101, PAK1-mediated	Inhibition	Brain tumor^160^	Tumor development
	OPTN	S177, TBK1-meidated	Activation	Infectious disease^162^	Salmonella clearance
	OPTN	S526, p85β-mediated	Inhibition	Tumor^165^	Tumor development
	ATG4B	S383, MST4-mediated	Activation	Glioblastoma^169^	Tumor development
	FOXO3a	S318, S321, CK1α-mediated	Inhibition	Multiple cancer^171^	Tumor suppression
	FOXO3a	S253, AKT-mediated	Activation	NSCLC^172^	Tumor suppression
	Beclin1	PP2A-mediated	Activation	Neurodegeneration, tumor^173–175^	Disease progress ↓
	AMPKα	T172, PP2A-mediated	Activation	ischemia/reperfusion^176^	Dysfunctional mitochondria clearance

### Acetylation

HDAC- and HAT-mediated TF acetylation at the post-translational level is at least one mechanism by which autophagy is transcriptionally elicited/muted. Whether TF acetylation promotes or inhibits transcriptional activity remains controversial and is likely dependent on specific acetylation sites. For example, p53 acetylation enhances its transcriptional activity,^110^ while FOXO1 acetylation decreases its transcriptional activity.^111^ TFEB acetylation at K91, K103, K116, and K430 indicates increased transcriptional activity.^112^ Conversely, GCN5-catalyzed K274 and K279 acetylation disrupts their target gene binding.^113^ In cancer cells with HDAC inhibition, enhanced TFEB acetylation at K91, K103, K116, and K430 markedly transcriptionally activates the expression of its target genes related to autophagy and lysosomal biogenesis and induces cell death.^112^ GCN5-catalyzed K274 and K279 acetylation of TFEB impedes lysosome formation and further the clearance of Tau protein aggregates, exacerbating the neurodegenerative phenotypes.^113^

Tip60-mediated p53 acetylation at K120, collaborating with PIASy-mediated K386 sumoylation, functions as a death signal to promote p53 cellular accumulation and autophagy.^114^ In addition, TFAM knockdown in tumor cells increases NAD(+)/NADH ratio and leads to SIRT1 upregulation to deacetylate p53 at K382 sites, which disturbs autophagy via weakening transcriptional regulation of the PISD enhancer.^115^ IKKα plays a cytoprotective role when treating human hepatoma cells with the antitumor therapeutic reagent arsenite. Interestingly, it triggers a feedback loop of autophagy-dependent degradation through p53 acetylation and thus contributes to arsenite-induced apoptosis. During this process, checkpoint kinase 1 (CHK1) activation phosphorylates p53 and undergoes p300/CBP-mediated acetylation at K373 and/or K382, triggering autophagy and autophagy-mediated IKKα degradation.^116^ Furthermore, SIRT1-mediated p53 deacetylation promotes autophagy and alleviates sepsis-induced acute kidney injury.^117^

HDACIs can induce autophagy through FOXO1-dependent pathways, whereas autophagy inhibition markedly enhances HDACI-mediated cell death.^118,119^ Acetylated FOXO1 may be responsible for HDAC-mediated autophagy regulation. When oxidative stress or serum starvation occurs, cytoplasmic FOXO1 is acetylated at K262, K265, and K274 via SIRT2 dissociation, facilitating the interaction between cytosolic FOXO1 and ATG7 and the autophagic process, leading to tumor suppression in an autophagy-dependent manner.^15,120^ In response to Angiotensin II stimuli, HDAC4 expression is upregulated in vascular endothelial cells, which consequently deacetylates FOXO3a to transcriptionally activate autophagy, and therefore leads to vascular inflammation.^121^

The acetylation status of a set of fundamental autophagy-related machineries has been well identified and proven to be involved in autophagosome and autolysosome formation.^122^ Including Beclin1, LC3, p62, and STX17, these critical autophagic effectors function distinctively in the autophagy process, depending on their acetylation status. By affecting spatial structure-dependent interactions with other autophagic cofactors, acetylated and deacetylated proteins represent either non-autophagic (acetylated LC3 and Beclin1) or active autophagic forms (deacetylated LC3, acetylated p62 and STX17).^123^

Typically, Beclin1 acetylation is emblematic of autophagy repression. In particular, p300- and SIRT1-regulated Beclin1 acetylation at the K430 and K437 sites is associated with tumor development,^124^ sensitivity impairment to anticancer drugs^125^ and sepsis-induced acute kidney injury/cardiac dysfunction.^126,127^ SIRT1 or SIRT6 overexpression deacetylates Beclin1 and activates autophagy. Activated autophagy has been shown to promote the epithelial-mesenchymal transition (EMT) of cancer cells.^128,129^ This adverse effect takes advantage of the autophagic protein degradation pathway to accelerate epithelial-specific protein reduction. Similarly, LC3 can also be a direct substrate of SIRT1. By deacetylating LC3, the interplay between LC3 and ATG3 is strengthened, assisting autophagy-p62-mediated phosphatase and tensin homolog (PTEN) degradation and accelerating liver cancer occurrence.^129^ Autophagy prompted by stabilized SIRT1-induced ATG5 and ATG7 protein deacetylation causes chemoresistance in liver cancer cells and protect vascular endothelial cells from oxidant-induced cell injury.^130^

Adiposity is confirmed to be associated with sex difference, SIRT1 and autophagy. Mechanistically, estrogen receptor alpha induced SIRT1 expression, which then deacetylates and activates AKT and STAT3, resulting in suppression of autophagy and adipogenesis via mTOR-ULK1 and p55 cascades.^131^ Moreover, HDAC4-STAT1 signaling-regulating autophagy inhibition is partially responsible for podocyte injury during diabetic neuropathy, in which STAT1 is deacetylated by HDAC4 to promote its phosphorylation and activation, which then translocates into the nucleus to induce gene expression, leading to the induction of inflammation and apoptosis and the suppression of autophagy.^132^

Cytoskeleton proteins, such as α-tubulin and cortactin, are quite essential for autophagy vesicular trafficking and autophagy-lysosome fusion. HDAC6 promotes autophagy by recruiting a cortactin-dependent, actin-remodeling machinery, which in turn assembles an F-actin network that stimulates autophagosome-lysosome fusion and substrate degradation. HDAC6 deficiency or deregulation results in autophagy dysfunction, and thus retardation of protein aggregates degradation.^133^

**Fig. 5:**
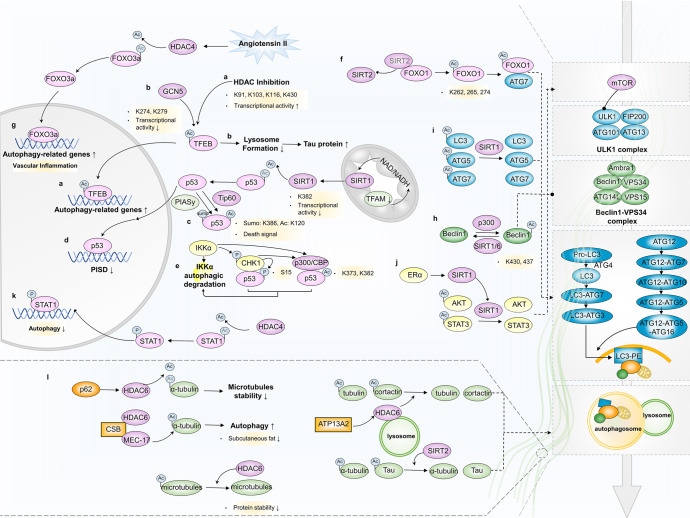
Acetylation modification and autophagy regulation. **a** TFEB acetylation at K91, K103, K116, and K430 markedly activates the expression of its target genes related to autophagy and lysosomal biogenesis and induces cell death. **b** GCN5-catalyzed K274 and K279 acetylation of TFEB impedes lysosome formation and the clearance of Tau protein aggreagates. **c** Tip60-mediated p53 acetylation at K120 and K386 sumoylation function as a death signal to promote p53 cellular accumulation and autophagy. **d** P53 acetylation at K382 sites affects autophagy levels via the transcriptional regulation of the PISD enhancer. **e** P53 is phosphorylated by CHK1 activation and undergoes p300/CEP-mediated acetylation at the K373 and/or K382 sites, which triggers autophagy and autophagy-mediated IKKα degradation. **f** Cytoplasmic FOXO1 is acetylated at K262, K265, and K274 via SIRT2 dissociation, facilitating the interaction between cytosolic FOXO1 and ATG7 and the autophagic process. **g** Upregulated HDAC4 deacetylates FOXO3a to transcriptionally activate autophagy. **h** P300 and SIRT1/6-regulated acetylation of Beclin1 at the K430 and K437 sites. **i** SIRT1 overexpression deacetylates LC3, ATG5 and ATG7 and activates autophagy. **j** ERα-induced SIRT1 expression deacetylates and activates AKT and STAT3, resulting in suppression of autophagy via mTOR-ULK1 and p55 cascade. **k** HDAC4-STAT1 signaling-regulating autophagy inhibition. (l) HDAC6-mediated cytoskeleton proteins acetylation regulation

ATP13A2 mutations cause Kufor-Rakeb syndrome in juvenile-onset atypical Parkinson’s disease. HDAC6 is recruited by ATP13A2 to lysosome and deacetylates cortactin and tubulin to promote autophagosome-lysosome fusion and protein aggregates degradation.^134^ HDAC6 upregulation after spinal cord injury induces microtubules deacetylation and reduced stability, leading to autophagy inhibition and injury occurrence.^135^ In prostate cancer, p62 increases HDAC6 levels and reduces the acetylation of α-tubulin and the stability of microtubules, causing autophagy flux impairment and EMT.^136^ Cockayne syndrome group B (CSB) protein directly interacts with HDAC6 and acetyltransferase MEC-17 to antagonize the deacetylation of α-tubulin by HDAC6, leading to autophagy restoration and rescuing loss of subcutaneous fat.^137^ (Fig. 5). It is noteworthy that, in addition to HDAC6-medited cytoskeleton proteins acetylation, SIRT2 has also involved in this process, which regulates α-tubulin and Tau acetylation to affect autophagy vesicular traffic and cargo clearance in several neurological disorders, including Parkinson’s disease and Alzheimer’s disease.^138^

### Ubiquitination

**Fig. 6:**
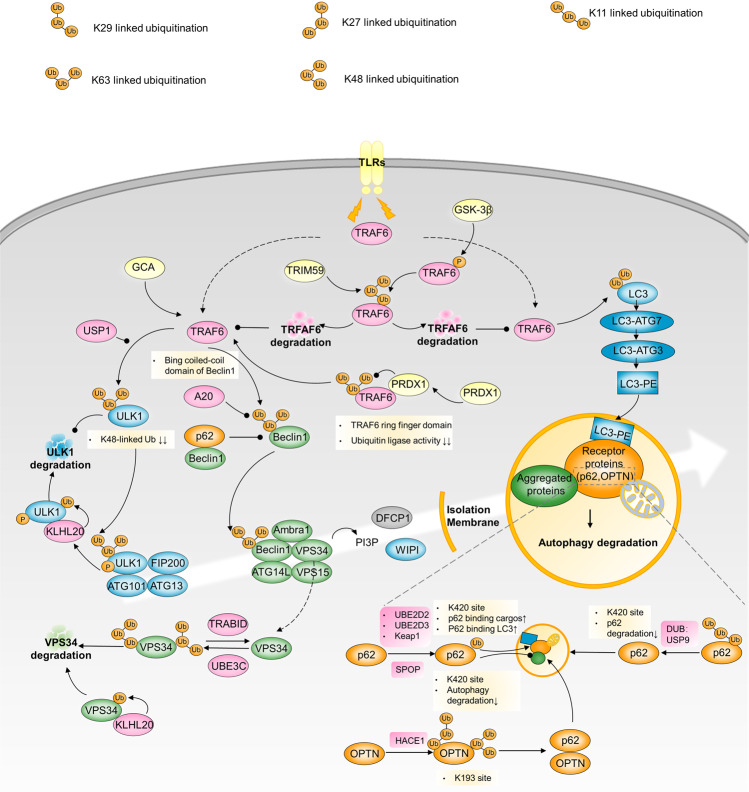
Ubiquitination and autophagy regulation. GCA triggers TRAF6 activity to induce K63-linked ULK1 ubiquitination. ULK1 deubiquitination by USP1 is required after TRAF6-induced ULK1 ubiquitination. Upon autophagy induction, ULK1 autophosphorylation facilitates its recruitment to KLHL20 for ubiquitination and proteolysis, which restrains the amplitude and duration of autophagy. TLR-mediated signaling can induce autophagy via the association of TRAF6 with the coiled-coil domain of Beclin1 and Beclin1 ubiquitination. A20 reduces the extent of K63-linked Beclin1 ubiquitination. PRDX1 binds to TRAF6 to inhibit K63-linked-ubiquitination of TRAF6, leading to reduced TRAF6 ubiquitin-ligase activity, which fails to activate Beclin1. KLHL20, UBE3C and TRABID reciprocally regulate K29/48-branched ubiquitination of VPS34, mediating its proteasome degradation. TRAF6 polyubiquitinates LC3B and promotes LC3B-ATG7 complex formation to drive selective autophagic CTNNB1 degradation. HACE1 ubiquitinates OPTN with K27 and K48 ubiquitin linkages and on multiple lysine residues of OPTN containing K193. OPTN ubiquitination at K193 promotes the interaction with p62 to activate autophagy. USP8 deubiquitinates p62 by removing the K11-linked ubiquitin chains from p62, and the deubiquitinated site is principally located at K420 site, which leads to p62 reduced degradation and autophagy flux. p62 can be also ubiquitinated by Keap1/Cullin3 to promote p62’s association with LC3 and autophagy

Ubiquitination is a reversible enzymatic process of ubiquitin linkage and removal catalyzed by a succession of ubiquitin ligases (including E1, E2, and E3) and deubiquitinating enzymes. Mono- or polyubiquitination, K48- or K63-linked ubiquitination, and various modified residues on autophagy-related components dictate whether to inhibit or activate autophagy^139^ (Fig. 6).

### ULK1 ubiquitination

TNF receptor-associated factor 6 (TRAF6) and ubiquitin specific peptidase 1 (USP1) are ubiquitinases and deubiquitinases that deal with unc-51-like autophagy activating kinase 1 **(**ULK1) ubiquitin modifications. In imatinib-resistant chronic-phase chronic myeloid leukemia (CML), upregulated GCA triggers TRAF6 ubiquitinase activity to induce K63-linked ULK1 ubiquitination and reduces K48-linked ubiquitination. This results in ULK1 stabilization and pro-survival autophagy activation.^140^ For robust post-initiation autophagy, ULK1 deubiquitination by USP1 is required after TRAF6-induced ULK1 ubiquitination, promoting breast cancer cell survival and proliferation. Upon USP1 dysfunction or depletion, canonical autophagy cannot be fully activated; conversely, it turns out to be uncanonical autophagy, only using part of the autophagic machinery for cargo clearance. USP1 inhibition may potentiate the death of autophagy-competent cancer cells.^141^ In addition to be involved in autophagy initiation, ULK1 is also a critical autophagy brake. Upon autophagy induction, ULK1 autophosphorylation facilitates its recruitment to KLHL20 for ubiquitination and proteolysis, which restrains the amplitude and duration of autophagy. Impairment of KLHL20-mediated regulation of autophagy dynamics potentiates starvation-induced cell death and aggravates diabetes-associated muscle atrophy.^142^

### TRAF6-Beclin1

TRAF6 ubiquitinates Beclin1 generally activated by K63-linked ubiquitination. Various upstream signaling molecules influence TRAF6-Beclin1. Toll-like receptor (TLR)-mediated signaling can induce autophagy via the association of TRAF6 with the coiled-coil domain of Beclin1 and ubiquitination of Beclin1. The deubiquitinating enzyme A20 reduces the extent of TLR signaling-induced K63-linked Beclin1 ubiquitination.^143^ Interestingly, autophagy induction contributes to TLR4- and TLR3-triggered progression of lung cancer cells through TRAF6 ubiquitination.^144^ In contrast, peroxiredoxin 1 (PRDX1), an antioxidant protein, counteracts TRAF6-dependent Beclin1 ubiquitination induced by TLR4 stimulation. Mechanistically, PRDX1 binds to the ring finger domain of TRAF6 to inhibit its own K63-linked-ubiquitination of TRAF6, leading to reduced TRAF6 ubiquitin ligase activity and Beclin1 inactivation.^145^ Similarly, p62 can block the TRAF6-Beclin1 combination and catalytic processes by competitively interacting with Beclin1.^146^ Another non-negligible example of TRAF6-Beclin1 axis regulation involves tripartite motif-containing 59 (TRIM59), a tripartite motif protein with potential oncogenic activity. In NSCLC cells, TRIM59 expression is reciprocally correlated with Beclin1 expression and basal autophagy levels. TRIM59 repress Beclin1 transcription by negatively regulating the NF-κB pathway, it also eliminates active ubiquitinated Beclin1 for TRIM59 functions as an E3 ligase that mediates K48-linked TRAF6 ubiquitination and promotes proteasomal TRAF6 degradation.^147^

### VPS34 ubiquitination

Ubiquitin ligase UBE3C and deubiquitinating enzyme TRABID have been found to reciprocally regulate K29/K48-branched ubiquitination of VPS34. This ubiquitination enhances proteasome-dependent degradation of VPS34, thereby suppressing autophagosome formation and maturation. TRABID is downregulated during the pathogenesis of steatosis, which decreases VPS34 stability and results in lipid metabolism disturbance.^148^ KLHL20 ubiquitin ligase can be recruited to VPS34 complex to mediates VPS34, Becline1, ATG14 ubiquitination and degradation. Impairment of KLHL20-mediated regulation of autophagy dynamics aggravates diabetes-associated muscle atrophy.^142^

### TRAF6-LC3B

Aberrant CTNNB1 signaling frequently occurs in cancers, particularly colorectal cancer (CRC). TRAF6 can polyubiquitinate LC3B and promote LC3B-ATG7 complex formation to drive selective autophagic CTNNB1 degradation, beneficial for inhibiting cancer metastasis.^149^ However, in most clinical CRC, TRAF6 is phosphorylated at Thr266 by glycogen synthase kinase 3 beta (GSK3β), leading to increased K48-linked polyubiquitination and degradation, thereby inhibiting autophagy-dependent CTNNB1 reduction.^149^

### HACE1-OPTN

As a tumor suppressor, ubiquitin ligase HACE1 ubiquitinates optineurin (OPTN) with K27 and K48 ubiquitin linkages and on multiple lysine residues of OPTN containing K193. OPTN ubiquitination at K193 promotes p62 interactions to activate autophagy, accelerating damaged protein degradation and hampering tumorigenicity.^139^

### p62 ubiquitination

Ubiquitin stress is an inducer of p62 ubiquitination catalyzed by E2 ubiquitin conjugating enzymes UBE2D2 and UBE2D3. Ubiquitylation of p62 disrupts dimerization of the UBA domain of p62, liberating its ability to recognize polyubiquitylated cargoes for selective autophagy.^150^ Moreover, p62 can be also ubiquitinated by Keap1/Cullin3, with the modified site at K420 within its UBA domain to promote p62’s association with LC3 and autophagy.^151^ SPOP mutation is frequently mutated in prostate cancer. Different from above ubiquitin-induced p62 degradation and autophagy flux, the gene encoding the E3 ubiquitin ligase substrate-binding adapter SPOP leads to non-degradative ubiquitination of p62 at K420 and thus autophagy suppression.^152^ Conversely, USP8 has been reported to directly deubiquitinate p62. It preferentially removes the K11-linked ubiquitin chains from p62, and the deubiquitinated site is principally located at K420 site, which leads to p62 reduced degradation and autophagy flux.^153^

### Phosphorylation

**Fig. 7:**
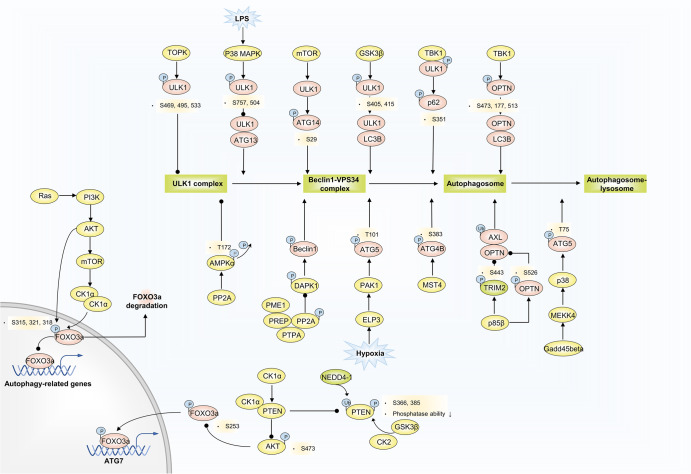
Phosphorylation and autophagy regulation. TOPK phosphorylates ULK1 at Ser469, Ser495, and Ser533, decreasing ULK1 activity and stability. LPS-activated p38 MAPK phosphorylates ULK1 at S757 and S504, preventing it from binding to ATG13, and reduced autophagy in microglia. GS3Kβ phosphorylates ULK1 at S405 and S415 sites, and thus promotes ULK1 and LC3B interaction. Activated ULK1 phosphorylates p62 and ATG14 to accelerate autophagy flux. Gadd45beta-MEKK4 pathway specifically directs p38 to autophagosomes, and p38 catalyzes phosphorylation of ATG5 at T75, resulting in an accumulation of autophagosomes through inhibition of lysosome fusion. ELP3 enhances PAK1 activity, thereby leading to ATG5 phosphorylation at T101. ATG4B is a direct MST4 substrate. MST4 phosphorylates ATG4B at S383 and contributes to increased autophagy activity. TRIM2 selectively ubiquitinates AXL to promote its autophagic degradation due to OPTN binding to the polyubiquitin chain of AXL. p85β mediates the phosphorylation of TRIM2 at the S443 residue and OPTN at S526, contributing to inhibited AXL degradation. Ras/PI3K/AKT/mTOR/CK1α/FOXO3 pathway limits an autophagy flux. CK1α/PTEN/AKT/FOXO3/ATG7 route excites autophagy. Polyubiquitination catalyzed by NEDD4-1 and phosphorylation by GSK3β and CK2 at the PTEN C-terminal reduce PTEN protein stability and inactivate phosphatase activity, activated PTEN blocks AKT phosphorylation at S473, subsequently inducing activated FOXO3a phosphorylation at S253. PP2A-DAPK1-Beclin1 and PP2A-AMPKα phosphorylation signaling pathways are involved in disease-related autophagy regulation

Phosphorylation is one of the most ubiquitous PTMs and is antagonistically orchestrated by a set of protein kinase and protein phosphatase families. Protein phosphorylation is a fundamental step in signal transduction. Over the past few years, extensive efforts have been made to explore crisscross signaling transduction pathways to manipulate autophagy. Direct phosphorylation events of an array of autophagy-relevant components are fairly critical for autophagy regulation, some of which even influence multiple disease-related biological processes (Fig.7).

### ULK1 phosphorylation

ULK1 protein contains quite rich phosphorylation modified sites, through which modulates autophagy inhibition or activation in response to various environmental conditions, engaging in different autophagy stages. In neuroinflammatory diseases, LPS-induced p38 MAPK activation phosphorylates ULK1 at S757 and S504, preventing it from binding to the downstream effector ATG13, and reduced autophagy in microglia.^154^ T-LAK cell-originated protein kinase (TOPK), an oncokinase, could directly bind to and phosphorylate ULK1 at Ser469, Ser495, and Ser533, decreasing ULK1 activity and stability. ULK1 Ser469, Ser495, and Ser533 mutations induce autophagy initiation and enhance the sensitivity of glioma cells to temozolomide (TMZ).^155^ High phosphorylation levels of ULK1 at S405 and S415 sites were observed in human pancreatic cancer cells. This phosphorylation situation is mediated by GSK3β and thus promote the interaction of ULK1 and MAP1LC3B to exhibit high levels of autophagy.^156^

ULK1, as a serine/threonine kinase, is also able to be implicated in the phosphorylation of downstream autophagic effector proteins. TBK1 coordinates with ULK1 to promote concerted phosphorylation of autophagy receptor SQSTM1/p62 at S351 site within the UBA domain. In amyotrophic lateral sclerosis (ALS) and frontotemporal lobar degeneration (FTLD), mutant TBK1 reduces SQSTM1/p62 phosphorylation and compromises cargo binding and clearance, responsible for related neurotoxicity.^157^ It has found that ULK1 can regulate ATP14 phosphorylation, which displays a special role in the context of Huntington’s disease. ULK1 phosphorylates ATG14 at S29 in an mTOR-dependent manner, and this phosphorylation critically regulates ATG14-VPS lipid kinase activity to control autophagy level for the clearance of polyglutamine disease protein.^158^

### ATG5 phosphorylation

ATG5 phosphorylation at T75 site is a representative marker of inhibitory autophagy. Gadd45beta-MEKK4-p38 pathway is one of the regulatory mechanisms responsible for this phosphorylation. Gadd45beta-MEKK4 pathway specifically directs p38 to autophagosomes, and then p38 catalyzes phosphorylation of ATG5 at T75, resulting in an accumulation of autophagosomes through inhibition of lysosome fusion.^159^ Furthermore, in brain tumor, the oncogenic role of PAK1 is mainly associated with autophagy. Under hypoxia condition, ELP3 enhances PAK1 activity, thereby leading to ATG5 phosphorylation at T101. This event not only protects ATG5 from ubiquitination-dependent degradation but also increases the affinity between the ATG12-ATG5 complex and ATG16L1.^160^

### OPTN phosphorylation

TBK1-meidated phosphorylation of OPTN is functional in selective autophagy of damaged mitochondria and Salmonella. TBK1 phosphorylates OPTN’s UBAN domain at S473, thereby expanding the binding capacity of OPTN to diverse Ub chains. Meanwhile, phosphorylation of S177 and S513 promotes recruitment and retention of OPTN/TBK1 on ubiquitinated, damaged mitochondria.^161^ Moreover, TBK1 can also phosphorylate OPTN at S177 site, which helps to enhance LC3 binding affinity and autophagic clearance of cytosolic salmonella.^162^ p85β, encoded by PI3KR2, is the p85 regulatory subunit of PI3K and is frequently amplified in cancers. The receptor tyrosine kinase AXL is an upstream PI3K regulator. In multiple cancer cells, AXL, an indicator of malignant phenotypes and poor prognosis, is upregulated but is rarely detected as genetically aberrant.^163,164^ The Ubiquitinase TRIM2 selectively ubiquitinates AXL to promote autophagic degradation due to OPTN binding to the polyubiquitin chain of AXL. However, p85β mediates the phosphorylation of TRIM2 at residue S443 and OPTN at residue S526, thereby inhibiting AXL degradation and activating AXL oncogenic signaling.^165^ However, how p85β regulates TRIM2 and OPTN phosphorylation remains to be elucidated, and this will be more conducive to further understanding the autophagic control mechanism in the AXL/p85β context.

### ATG4B phosphorylation

ATG4B is an intriguing ATG that has been reported to have tumorigenic functions and is regarded as a potential therapeutic target and prognostic marker.^166,167^ As a member of the mammalian sterile20-like (STE) serine/threonine kinase (STK) family, MST4 is prone to promoting tumor development through its phosphorylation activity (e.g., activating the tumor-promoting p-ERK pathway^168^). ATG4B is a direct substrate of MST4. MST4-directed ATG4B phosphorylation at S383, but not at other described phosphorylation sites, contributes to increased autophagy activity, consequently facilitating tumorigenesis and drug resistance to RT in glioblastomas.^169^ Interestingly, ATG4B phosphorylation can also be independent of autophagic flux to perform tumorigenic functions. Instead of affecting autophagy flux, AKT-mediated ATG4B phosphorylation at residue S34 aims to repress mitochondrial activity and enhance the Warburg effect in HCC cells through mitochondrial ATG4B translocation and subsequent inhibition of mitochondrial respiration-related F_1_Fo-ATP synthase.^170^

### CK1α mediated phosphorylation

Casein kinase 1 alpha (CK1α) belongs to a family of highly conserved monomeric Ser/Thr kinases, engaging in various biological functions, including membrane trafficking, cell cycle, cell metabolism, apoptosis, and autophagy. Focusing on autophagy regulation, we observed that the effects of CK1α were two-sided.

In one scenario, the Ras/PI3K/AKT/mTOR/CK1α/FOXO3 pathway limits autophagic flux. Oncogenic Ras-driven cancer cells exhibit elevated basal autophagy to cope with the pressure of rapid growth. CK1α has been found to be a negative regulator in oncogenic RAS-induced autophagic levels. Theoretically, oncogenic Ras, via its downstream PI3K/AKT/mTOR effector pathway, increases CK1α protein abundance. Later, elevated CK1α additionally phosphorylates already S315 site-phosphorylated FOXO3a at S318 and S321 to destabilize FOXO3a. FOXO3a is a positive TF in ATG regulation, increasing the expression of LC3B, ATG12, BNIP3, and others. Thus, unstable and null FOXO3 induced by CK1α impedes the expression of a cohort of ATGs and limits the autophagic recycling of nutrients.^171^

In another scenario, the CK1α/PTEN/AKT/FOXO3/ATG7 pathway excites autophagy. Loss of PTEN function is a frequent event in cancers owing to post-transcriptional and PTMs. At the PTEN C-terminal, polyubiquitination catalyzed by NEDD4-1 is associated with reduced protein stability, and phosphorylation by GSK3β and CK2 is linked to inactive phosphatase activity. CK1α, as a PTEN-interacting partner, strongly interacts with PTEN to antagonize NEDD4-1 induced PTEN polyubiquitination and competitively abrogate PTEN phosphorylation, especially at T366 and S385, which are the main sites catalyzed by GSK3β and CK2. Concomitantly, functional PTEN reduces AKT phosphorylation at S473, a signal of AKT inhibition, followed by specific FOXO3a phosphorylation at S253. Finally, ATG7, but not LC3B or ATG5, is exclusively transactivated by FOXO3a to induce autophagy, leading to NSCLC cell growth suppression.^172^

### PP2A-involved phosphorylation

Being involved in multiple autophagy-related signaling transduction pathways, protein phosphatase 2A (PP2A) is in a close correlation with the development of multiple diseases. PREP, a serine protease has been demonstrated to interact with PP2A and its endogenous inhibitor PME1 and activator PTPA, thus adjusting its activity and levels of PP2A. PREP inhibition represses PP2A phosphorylation and increases its activity, leading to DAPK1 and Beclin1 phosphorylation, and therefore autophagy induction. Several neurodegenerative diseases and cancers are connected with lower PP2A activity and PP2A activators may manifest great potential as drug therapy.^173–175^ After ischemia/reperfusion, increased PP2A and decreased AMPKα phosphorylation at T172 has been observed, attributing to acute kidney injury. Mechanistically, ischemia/reperfusion-triggered PP2A dephosphorylates T172-AMPKα, which decreases AMPK activity, suppresses ULK1-mediated autophagy, and thus impedes dysfunctional mitochondria clearance.^176^ Targeting PP2A-AMPK axis may be an effective therapeutic method to relieve cell injury due to abnormal autophagy dysfunction. As reported, GNMT deficiency in steatotic patients elevates the levels of methionine and SAMe, which accounts for PP2A methylation and its activity enhancement, leading to impaired autophagy flux and aggravating liver steatosis. Employing a methionine deficient diet normalizes the methylation capacity and PP2A methylation levels, benefiting autophagy restoration and lipid metabolism.^177^

### Crosstalk between phosphorylation and ubiquitination in autophagy regulation

**Fig. 8:**
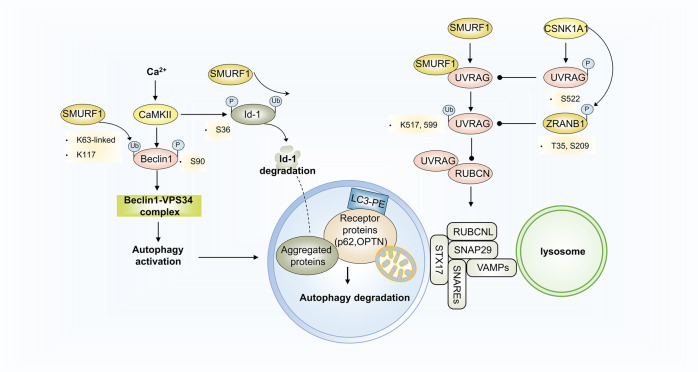
Crosstalk between phosphorylation and ubiquitination in autophagy regulation. SMURF1 forms a complex with UVRAG by binding to the PPxf motif and catalyzes K29- and K33-linked polyubiquitination of UVRAG at the K517 and K599 residues. Ubiquitinated UVRAG blocks RUBCN’s approach to the UVRAG-containing PIK3C3 complex, inhibiting the negative effect of RUBCN and promoting autophagosome maturation. CSNK1A1-mediated UVRAG phosphorylation at S522 disrupts the binding of SMURF1 to UVRAG and reverses UVRAG ubiquitination-mediated autophagosome maturation. CaMKII phosphorylates Beclin1 at S90 and subsequently increases TRAF6-catalyzed Beclin1 K63-ubiquitination, collectively contributing to autophagy induction for K63-linked ubiquitylated Id-1/2 degradation

SMURF1 is a HECT-type E3 ubiquitin ligase that regulates various biological signaling networks and is involved in different diseases and disorders. However, studies on autophagy modulation with SMURF1 engagement and mediation are limited. Casein kinase (CSNK) isoforms have been identified as constitutive autophagy-regulating kinases.^178^ Recently, a fascinating study verified that SMURF1 could form a complex with UVRAG by binding to the PPxf motif, and catalyze K29- and K33-linked UVRAG polyubiquitination at residues K517 and K599.^179^ Ubiquitinated UVRAG, instead of inducing ubiquitination-mediated protein degradation, blocks RUBCN’s approach to the UVRAG-containing PIK3C3 complex, which inhibits the negative effects of RUBCN and promotes autophagosome maturation.^179,180^ Nevertheless, CSNK1A1-mediated UVRAG phosphorylation at S522 disrupts SMURF1-UVRAG binding and reverses UVRAG ubiquitination-mediated autophagosome maturation.^179^ UVRAG K29- and K33-linked ubiquitination or prevention of UVRAG (S522) phosphorylation facilitates autophagy-dependent EGFR degradation and inhibits HCC growth,^179^ indicating a potential therapeutic strategy targeting post-translationally modified autophagy-related proteins (Fig. 8a).

Moreover, calcium/calmodulin-dependent protein kinase II (CaMKII) was found to influence post-translational autophagy regulation. CaMKII is a serine/threonine-protein kinase activated by Ca^2+^ and calmodulin (CaM). Notably, the Ca^2 +^/CaMKII-regulating signaling pathway that triggers or inhibits autophagy varies and is mainly dependent on specific cell types and contexts. For instance, the BAFF treatment-activated Ca^2+^-CaMKII-dependent Akt/mTOR signaling pathway inhibits autophagy and promotes cell proliferation and survival in normal and neoplastic B-lymphoid cells,^181^ whereas CaMKII directly targeting Beclin1 can induce autophagy to promote solid tumor cell differentiation. In the latter regulatory mechanism, CaMKII directly phosphorylates Beclin1 at S90 and subsequently increases TRAF6-catalyzed K63-ubiquitination of Beclin1, collectively contributing to autophagy induction for K63-linked ubiquitylated Id-1/2 degradation^182^ (Fig. 8b).

## Epigenetic drugs and autophagy

Epigenetic compounds have become imperative tools for disease treatments, including treatment of cardiovascular disorder, infectious disease, and particularly tumor, which manifest potent therapeutic effects by eliciting apoptosis, cell cycle arrest, and differentiation (currently clinically applied epigenetic drugs are described and summarized elsewhere^27^). Although a few epigenetic drugs have showed great therapeutic effectiveness in clinical development and several have been officially approved by the FDA, the application of epigenetic drugs in disease treatment remains limited and still cell or disease type-dependent. Current studies have been investigating the potential therapeutic effectiveness of these drugs in multiple disease. However, it has been found that therapeutic effectiveness varies a lot in different contexts, and relevant molecular events are also vague and remain to be vividly elucidated. Autophagy is a multifaceted functional event that promotes cytoprotective or cell-damage, antitumor or tumorigenic activities. Indeed, numerous scientific studies have indicated the dual autophagic effects that is associated with diverse therapeutic consequences of epigenetic drug treatment, either serving as a synergistic mechanism to strengthen therapeutic response or an antagonistic mechanism to induce drug resistance and loss of effectiveness. Here, we summarize the possible molecular mechanisms by which autophagy distinctively affects the therapeutic consequences of epigenetic compounds, to provide novel insights into the combined utilization of epigenetic drugs and autophagy activators or inhibitors (Table 3).

**Table 3:** Application of epigenetic drugs in autophagy-related diseases

Drugs		Disease types	Autophagy activation or inhibition	The effect on diseases	Relevant mechanism
DNMT inhibitors	Se-allylselenocysteine	Colorectal cancer	Activation	Tumor suppression^183^	PCDH17 expression upregulation through hypomethylation of its promoter
	Decitabine	Acute myeloid leukemia	Activation	Tumor suppression^184^	TIGAR downregulation
		High-risk myelodysplastic syndrome	Activation	Tumor suppression^185^	FOXO3A reactivation
	5-Azacitidine	High-risk myelodysplastic syndrome	Activation	Tumor pro-survival^186,187^	–
Histone deacetylase inhibitors	SAHA	Cancer	Activation	Tumor pro-survival^188^	p53 degradation
		Breast cancer	Activation	Tumor pro-survival^189^	Targeting HDAC3 and HDAC6, survivin and XIAP downregulation
	TSA	–	Activation	Tumor pro-survival^192,193^	mTOR blockage, transcriptional activity activation of FOXO1
		Cardiopathy	Inhibition	cardiac hypertrophy↓^194^	–
	Salermide	Lung cancer	Activation	Tumor pro-survival^195^	SIRT11/2 repression, HSPA5 acetylation, ATF4 and DDIT4 activation, mTOR pathway inhibition
	VPA	Gastric cancer	Activation	Tumor suppression^196^	HDAC1/PTEN/AKT signaling pathway
		Myocardiopathy	Activation	myocardial dysfunctions↑^198^	PTEN/AKT/mTOR
	Spermidine	Hepatocellular cancer	Activation	Tumor suppression^199^	Nucleus translocation of HDAC4 and MAP1S acetylation
	Sulforaphane	Triple-negative breast cancer	Activation	Tumor suppression^200^	HDAC6 repression, PTEN acetylation
	TMU-35435	Triple negative breast cancer	Activation	Tumor suppression^201^	HDAC6 repression, misfolded protein aggregation
	MHY2256	Endometrial Cancer	Activation	Tumor suppression^202^	SIRT1 inhibition, p53 acetylation
	SAHA/TSA	kidney injury	Activation	Injury ↓^203^	AMPK/mTOR
	TSA	Sepsis-induced organ injury	Activation	Injury ↓^204^	M2 phenotype transformation of macrophage
BET inhibitors	JQ1	Spinal cord injury	Activation	Injury ↓^207^	AMPK/mTOR/ULK1
		Diabetic cardiomyopathy	Activation	Cardiac function ↑^208^	PINK1/Parkin
		Tumor	Activation	Tumor suppression^209^	FTH1 degradation, ferritinophagy
		Acute myeloid leukemia	Activation	Drug resistance^211^	AMPK-ULK1 pathway
		Ovarian cancer cells	Activation	Drug resistance^212^	Akt/mTOR pathway
	Compound 17c	Colorectal cancer	Activation	Tumor suppression, drug sensitivity increase^210^	BRD4-AMPK pathway, IL6-JAK-STAT signaling pathway
Histone methylase inhibitors	BIX01294	Multiple myeloma and glioblastoma	Activation	Tumor suppression^213–215^	mTOR/4EBP1 pathway, c-MYC reduction
		Head and neck squamous cell carcinoma	Activation	Tumor suppression^50^	DUSP4-dependent ERK-mTOR dephosphorylating pathway
	SH003	Gastric cancer	Activation	Tumor suppression^217^	IRE1/JNK/CHOP pathway
	Kaempferol	Gastric cancer	Activation	Tumor suppression^218^	PERK/ATF4/CHOP signaling pathway
	GSK343	Pancreatic cancer	Activation	Tumor suppression^219^	AKT/mTOR
	EPZ-011989	Epithelioid sarcoma	Activation	Tumor suppression^67^	HMGA2-mediated
	UNC1999	Aortic dissection	Activation	VSMCs function ↓^64^	MEK-ERK1/2 signaling pathway

### DNMT inhibitors

Se-allylselenocysteine (ASC) can decrease global DNA methylation levels by downregulating DNMT1 expression.^183^ In human CRC, ASC induces autophagic cell death through increased PCDH17 expression and reduces hypermethylation of its promoter in human CRC. Another DNMT inhibitor, decitabine (DAC), is widely prescribed for treating acute myeloid leukemia (AML) and is recommended for patients with high-risk myelodysplastic syndrome (MDS). Sustained exposure to DAC treatment leads to apoptotic cell death in AML by promoting differentiation, senescence, and autophagy,^184^ which may be due to the unmethylated role of DAC in downregulating TIGAR.^185^ In high-risk MDS patients, FOXO3A reactivation is responsible for DAC-induced autophagy, cell cycle arrest, and apoptosis. However, unlike cancer cell-adverse autophagy induced by the aforementioned drugs, autophagy appears to serve as a pro-survival pathway in MDS patients with long-term exposure to 5-Azacitidine.^186,187^ The novel autophagy inhibitor ROC-325 augments the antileukemic activity of azacytidine.^188^

### HDACIs

HDACIs can induce or inhibit autophagy. It has been reported that NF-kB hyperacetylation, p53 deficiency and acetylation, FOXO1 transcription, reactive oxygen species (ROS) accumulation, nuclear factor erythroid 2-related factor 2 (NRF2) and p21 upregulation, and mTOR inhibition might account for HDACI-induced autophagy.^189^

On the one hand, autophagy activation attenuates HDACI therapeutic effects and promotes cancer cell survival or sustained tissue damage. For example, suberoylanilide hydroxamic acid^168^ induces autophagy, accelerating mutated p53 degradation and assisting cancer cell survival.^190^ In breast cancer cells, SAHA targeting HDAC3 and HDAC6 elicits autophagy by downregulating survivin and XIAP gene transcription and inducing survivin protein acetylation and early nuclear translocation.^191^ Trichostatin A (TSA) strengthens autophagy levels by blocking the mTOR pathway and increasing FOXO1 transcriptional activity, whereas autophagy inhibition, such as using autophagy inhibitor chloroquine, can facilitate TSA-induced cell death and apoptosis.^192,193^ As an obligatory element, autophagy plays important role in pathological cardiac remodeling. TSA treatment suppresses autophagy via affecting HDAC1/2 activity and thus attenuates cardiac hypertrophy.^194^ Additionally, SIRT1/2 inhibition displays anticancer potential in a set of cancer types. However, in lung cancer cells, the SIRT1/2-specific inhibitor salermide upregulates pro-survival autophagy via HSPA5 acetylation and subsequent activation of activating transcription factor 4 (ATF4) and DNA damage inducible transcript 4 (DDIT4) to inhibit the mTOR signaling pathway.^195^

In contrast, autophagy accompanied by HDACIs manifests a synergistic therapeutic effect, such as enhancing cancer cell sensitivity to chemotherapy and alleviating cell injury. In gastric cancer, HDAC1/2 is overexpressed, displaying a positive correlation with poor prognosis. Valproic acid (VPA) treatment inhibits HDAC1/2 activity and induces autophagy through the HDAC1/PTEN/Akt signaling pathway, leading to increased apoptosis.^196^ VPA, combined with the mTOR inhibitor temsirolimus, synergistically inhibits cell growth and triggers autophagic cell death in the context of Burkitt leukemia/lymphoma.^197^ In addition, VPA-induced autophagy attenuates sepsis-induced myocardial dysfunction via HDAC1/3 inhibition and PTEN/AKT/mTOR pathway.^198^ Spermidine, a natural compound, induces autophagy by inhibiting EP300, which is primarily related to its cytoplasmic effects.^199^ In HCC treatment, spermidine promotes HDAC4 nuclear translocation to elevate cytoplasmic MAP1S acetylation, leading to autophagy induction and tumor development inhibition. Furthermore, sulforaphane, a natural histone deacetylase inhibitor, induced autophagy in triple-negative breast cancer. The underlying mechanism is associated with HDAC6 repression, which results in membrane translocation and PTEN acetylation.^200^ A novel HDACI, TMU-35435, has also been reported to suppress HDAC6 and cause misfolded protein aggregation, inducing autophagic cell death and enhancing radiation sensitivity in triple-negative breast cancer treatment.^201^ Additionally, MHY2256, a new synthetic histone deacetylase inhibitor, induces apoptosis and autophagic cell death via p53 acetylation and SIRT1 inhibition.^202^ Moreover, SAHA and TSA-induced autophagy via AMPK/mTOR pathway protects kidney injury from cisplatin treatment in proximal tubular cells.^203^ Interestingly, TSA can induce autophagy process in macrophage, modulate its M2 phenotype transformation to reduce sepsis-induced organ injury.^204^ These evidences demonstrating accessible treatment methods combining HDACIs and autophagy activators.

### Bromodomain and extra-terminal domain inhibitors

The BET family of proteins are epigenetic readers that recognize acetylated lysine residues on histone and non-histone proteins. Bromodomain-containing protein 4 (BRD4), a member of the BET family, has recently been shown to be involved in ATG expression transcriptional regulation. Under nutrient-sufficient conditions, BRD4 recognizes KAT8/hMOF-resultant histone H4K16 acetylation targets on the promoter regions of autophagy and lysosome genes. Subsequently, G9a is recruited to catalyze H3K9 demethylation and represses autophagy gene transcription. Upon nutrient deprivation, the histone deacetylase SIRT1 is released from its inhibitory molecule, CCAR2/DBC1, in an AMPK-dependent manner. Active SIRT1 then deacetylates H4K16, which in turn dissociates BRD4 from the promoter regions and leads to active autophagic transcription programs.^205,206^

As a BRD4 inhibitor, JQ1 has been shown to promote functional recovery from spinal cord injury by activating autophagy in an AMPK/mTOR/ULK1 pathway-dependent manner.^207^ Similarly, upregulation of BRD4 in diabetic mouse hearts inhibits PINK1/Parkin-mediated mitophagy, resulting in accumulation of damaged mitochondria and subsequent impairment of cardiac structure and function. JQ1 activates mitophagy via PINK1/Parkin, helping improve mitochondrial function and ameliorate diabetic cardiomyopathy.^208^ Also, JQ1 is able to induce ferroptosis, where ferritinophagy (a process characterized by increased autophagy and iron levels) is required to enhance cell death.^209^ In this process, JQ1-mediated autophagy degrades ferritin heavy chain 1 (FTH1), leading to enhanced intracellular iron and ROS levels. Upon autophagy inhibition, ferroptosis, at least in part, is limited in the context of JQ1 treatment. Furthermore, other molecular agents, such as selective BRD4/HDAC dual inhibitor compound 17c (inducing BRD4-AMPK-mediated autophagic cell death and suppressing the IL6-JAK-STAT signaling pathway that is related to drug resistance) and a novel small-molecule BRD4 inhibitor 9 f (FL-411) have been developed and discovered as potential BRD4 inhibitors that can induce autophagic cell death in different cancer models.^210^ However, in AML, JQ1-activated autophagy, which is AMPK-ULK1-mediated and mTOR signaling-independent, results in JQ1 resistance in AML stem cells.^211^ Additionally, Akt/mTOR-mediated JQ1-induced autophagy causes JQ1 resistance in ovarian cancer cells.^212^

### Histone methylase inhibitors

G9a inhibition can mechanistically trigger autophagy. In several cancer models, including multiple myelomas and glioblastomas, the G9a inhibitor BIX01294 targeting G9a induces cell proliferation inhibition and autophagy-associated apoptosis by inactivating the mTOR/4EBP1 pathway and transcriptionally reducing c-MYC levels.^213–215^ A genome-wide study illustrated that the interaction between MYC and G9a, with the MYC MBII region as the binding point, affects MYC’s capacity to drive transcriptional repression and tumorigenesis. Inhibiting G9a catalytic activity preferentially hinders MYC binding to repressed genes and shifts repressed loci with H3K9me2 enrichment to active epigenetic states.^216^ Abnormal G9a expression is also a survival advantage of head and neck squamous cell carcinoma. Instead of apoptosis or necrosis induction, pharmacological G9a suppression activates autophagic cell death mainly through the DUSP4-dependent ERK-mTOR dephosphorylation pathway.^50^ In addition, Two other potential antitumor agents, SH003 and kaempferol, have also been reported to efficiently inhibit G9a and induce autophagic cell death. The autophagy-associated tumor-inhibiting effects of SH003 and kaempferol appear to be a combination of G9a inhibition and endoplasmic reticulum (ER) stress, which involves the IRE1/JNK/CHOP and PERK/ATF4/CHOP signaling pathways.^217,218^

S-adenosyl-L-methionine (SAM)-competitive inhibitors is a major class of EZH2 inhibitors, such as EPZ005687, EI1, GSK126, UNC1999 and GSK343. In pancreatic cancer, GSK343 suppresses cell proliferation and induces apoptosis and autophagy via AKT/mTOR signaling pathway.^219^ In colorectal cancer, GSK343 and UNC1999 transcriptionally upregulated autophagy to promote cell death, but it is in an EZH2-independent manner.^220^ A comparative assessment of antitumor effects of drugs in epithelioid sarcoma indicated that autophagy induction is a possible survival mechanism in residual tumor cells following EZH2 inhibitor EPZ-011989 treatment, in which HMGA2 is a main player in this process.^67^ In contrast, in the progress of aortic dissection, UNC1999 employment to inhibit EZH2 activity appears to be detrimental for the function of vascular muscle cells (VSMCs), which is due to autophagic cell death exacerbation via MEK-ERK1/2 signaling pathway, highlighting from point to surface that the heterogeneity of outcomes of EZH2-induced autophagy in context-dependent diseases.^64^

## Drugs targeting phosphorylation modification and autophagy

Since phosphorylation is a fairly significant process in autophagic signaling transduction pathway, efforts have been made to seek for some chemical agents that could modulate the phosphorylation modification of critical autophagy-related components to specifically and efficiently activate or suppress autophagy (Table 3).

A cell-based screen has found that a potent and highly selective ULK1 kinase inhibitor, SBI-0206965, suppresses ULK1-mediated phosphorylation events by decreasing Beclin1 and VPS34 phosphorylation, upregulating autophagy level and inducing tumor cell apoptosis.^221^ PIKfyve kinase inhibitors, such as WX8, can disrupt lysosome function in autophagy and selectively kill certain cancer cells. Analysis of biochemical changes caused by PIKfyve kinase inhibitors reveals that resistant cancer cells contain higher level of p38MAPK protein and phosphorylation, which subsequently results in phosphorylation of lysosomal-associated membrane protein 2 (LAMP2) and compensatory autophagy function. Combined use of PIKfyve and p38MAPK inhibitors, WX8 and SB202190, synergistically blocks autophagy and markedly reduces the viability of multiple cancer cell types.^222^ Under nutrient-replete conditions, the vertebrate nonreceptor tyrosine kinase SRMS phosphorylates FKBP51, disrupts the FKBP51-PHLPP complex. This prevents PHLPP-mediated dephosphorylation of AKT, causing sustained AKT activation that inhibits autophagy and promotes tumor growth. SRMS kinase inhibition activates autophagy and inhibits tumor growth, and can be accomplished by using FDA-approved tyrosine kinase inhibitor Ibrutinib.^223^ In gastric cancer, Salidroside decreases the phosphorylation of PI3K/AKT/mTOR and induces apoptosis and protective autophagy. Treatment with the autophagy inhibitor chloroquine enhanced Salidroside-induced apoptosis.^224^ TGF-β-mediated super-activation of urethra fibroblasts contributes to the progression of traumatic urethral stricture. However, this kind of super-activation can be reversed by the use of a Rho-associated kinase inhibitor Fasudil and autophagy inhibitors. During this process, Fasudil is able to hamper TGF-β-induced super-activation by hampering autophagy in an AKT/mTOR pathway-dependent manner.^225^ It has reported that PREP suppresses autophagy via PP2A-DAPK1-Beclin1 signaling pathway in the progression of neurodegenerative disorders. Further, a small-molecule inhibitor KYP2407 has been screened out to stimulate autophagy and reduces the accumulation of α-SYN aggregates.^226^ Exendin-4 is the GLP1R agonist and stimulates autophagic flux in a setting of chronic nutrient excess, such as in type 2 diabetes. Mechanistically, RAPGEF4/EPAC2 and downstream calcium signaling contributes dominantly to activated autophagic flux by exendin-4. Instead of being dependent on AMPK and mTOR signaling, this pathway regulates PPP3/calcineurin and then dephosphorylates its downstream effector TFEB to upregulate transcription of autophagy-related genes.^227^ Analogously, the natural compound trehalose promotes autophagy and ameliorates neurodegenerative phenotype by inducing rapid and transient lysosomal enlargement and membrane permeabilization. This effect correlated with the calcium-dependent phosphatase PPP3/calcineurin activation, TFEB dephosphorylation and nuclear translocation^228^ (Table 4).

**Table 4:** Application of drugs targeting phosphorylation modification in autophagy-related diseases

Drugs targeting phosphorylation modification	Target	Disease types	Autophagy activation or inhibition	Drug effect on diseases	Relevant mechanism
SBI-0206965	ULK1	Tumor	Activation	Tumor suppression^221^	ULK1-Beclin1/VPS34
WX8 and SB202190	PIKfyve kinase and p38MAPK	Tumor	Inhibition	Tumor suppression^222^	LAMP2 phosphorylation↓
Ibrutinib	SRMS	Tumor	Activation	Tumor suppression^223^	SRMS-FKBP51-AKT
Salidroside	PI3K	Tumor	Activation	Tumor suppression^224^	PI3K-AKT-mTOR
Fasudil	–	Traumatic urethral stricture	Inhibition	Injury alleviation^225^	AKT-mTOR
KYP-2047	PREP	Parkinson’s disease	Activation	Injury alleviation^226^	PP2A-DAPK1-Beclin1
Exendin-4	GLP1R	Type 2 diabetes	Activation	Pancreatic β cells survival^227^	RAPGEF4/EPAC2--PPP3/calcineurin-TFEB
Trehalose	–	Neurodegeneration	Activation	Neurodegeneration amelioration^228^	PPP3/calcineurin-TFEB

## Conclusion

Autophagy can be aberrantly activated or inhibited during disease occurrence and progression, displaying cytoprotective or cell-damage, oncogenic or tumor-inhibiting functions. Although the multifactor and heterogeneity of disease-relevant autophagy requires further elucidation, systematic experimental studies across diverse disease types have revealed a wealth of regulatory mechanisms to help understand the reasons for aberrant alteration of autophagy level. This review focuses on epigenetic and post-translational modifications, including classic DNA methylation, histone methylation and acetylation, microRNAs and non-histone acetylation, ubiquitination, phosphorylation to integrate the multiplicity of chemically modified enzymes, related modified targets, and their modification sites, providing potential disease-specific autophagic biomarkers and predicting the development of novel clinical therapeutic targets. Importantly, this review also investigates the effects of autophagy, accompanied by the application of a series of epigenetic drugs and some phosphorylase inhibitors. The distinct consequences of autophagy induction or inhibition may potentiate an optimal combination of autophagy inducers or inhibitors with drug therapies. However, there are still various constrains remain to be further investigated and solved.

At present, epigenetic and post-translational regulation of autophagy may not be comprehensive. Research on many other emerging modifications, such as those modified on mRNA, including N^6^-methyladenosine (m^6^A) and N^1^-methyladenosine (m^1^A), histone or non-histone phosphorylation, glycosylation, and sumoylation are still have not been elucidated clearly. It indicates that these unknown modifications in autophagy regulation deserve an investigation, which will facilitate to further understand regulatory mechanisms and explore new molecular targets. Moreover, autophagy-relevant detection in humans remains difficult and vacant in clinical tests. Due to the scarcity of certain molecules that can specifically represent autophagy activation or inhibition, it highlights the importance of seeking for highly specific autophagic indicators and the development of new detection approaches. Additionally, although many experimental studies have demonstrated the efficacy of combined treatment of autophagy activators or inhibitors with chemotherapy, RT, and immunotherapy, very few studies have investigated the outcomes of treatment with autophagy activators or inhibitors combined with epigenetic therapies. Simultaneously, we should also pay attention to the specificity of autophagy activators and inhibitors that would possibly result in unexpected treatment effects and unsensitive response. Exploring and identifying small-molecule modulators targeting specific autophagic proteins could be a promising approach to solve sensitivity and effectiveness of autophagy-related drugs.

## Data Availability

All data generated or analyzed during this study are included in this published article and its supplementary information files.
